# Comparative Analysis of the Effectiveness of Three Immunization Strategies in Controlling Disease Outbreaks in Realistic Social Networks

**DOI:** 10.1371/journal.pone.0095911

**Published:** 2014-05-02

**Authors:** Zhijing Xu, Zhenghu Zu, Tao Zheng, Wendou Zhang, Qing Xu, Jinjie Liu

**Affiliations:** Center for Biosecurity Strategy Management, Beijing Institute of Biotechnology, Beijing, P. R. China; University of California Irvine, United States of America

## Abstract

The high incidence of emerging infectious diseases has highlighted the importance of effective immunization strategies, especially the stochastic algorithms based on local available network information. Present stochastic strategies are mainly evaluated based on classical network models, such as scale-free networks and small-world networks, and thus are insufficient. Three frequently referred stochastic immunization strategies—acquaintance immunization, community-bridge immunization, and ring vaccination—were analyzed in this work. The optimal immunization ratios for acquaintance immunization and community-bridge immunization strategies were investigated, and the effectiveness of these three strategies in controlling the spreading of epidemics were analyzed based on realistic social contact networks. The results show all the strategies have decreased the coverage of the epidemics compared to baseline scenario (no control measures). However the effectiveness of acquaintance immunization and community-bridge immunization are very limited, with acquaintance immunization slightly outperforming community-bridge immunization. Ring vaccination significantly outperforms acquaintance immunization and community-bridge immunization, and the sensitivity analysis shows it could be applied to controlling the epidemics with a wide infectivity spectrum. The effectiveness of several classical stochastic immunization strategies was evaluated based on realistic contact networks for the first time in this study. These results could have important significance for epidemic control research and practice.

## Introduction

Infectious diseases are diseases caused by pathogenic microorganisms, such as bacteria, viruses, parasites or fungi, which can be spread directly or indirectly from person to person. In recent years, newly emerging diseases have been identified with an unprecedented rate of one or more per year[1]. Meanwhile, highly developed transportation systems have intensified the large-scale spreading trend of the epidemics thus highlights the urgent demand for the research on effective control strategies. For many directly person-to-person transmitted diseases, such as HIV/AIDS, SARS and the influenza, network models could capture the specific contact patterns which could possibly lead to a successful transmission of the diseases between individuals and thus provide an effective way to forecast the potential epidemic dynamics and explore the intervention measures. In network models, the individuals are described as nodes (vertices) and the contacts between these individuals are described as edges (links). Seeded in a random selected node, the epidemic can be spread along the interconnections among vertices. The initial infected nodes will proceed to the infectious period after a short time after being infected, during when they could spread the infection to their contact nodes, and the newly infected nodes could then infect their linked vertices, and so on.

Effective control of infectious diseases requires quantitative comparisons of several interventions, such as quarantine, infection control precautions, case identification and isolation, and immunization. The success of an intervention depends on the infectivity of the diseases and the contact patterns of the population[2]. Network models define the detailed contact structure of the population, which would be conducive to the effectiveness assessment of different interventions, therefore they are widely applied to the study of immunization strategies. Several immunization strategies have been proposed based on network models. These strategies include both deterministic algorithms based on global network information, such as maximum-degree node immunization[3]–[7], maximum-betweenness node immunization[8], [9] and long-range travelers immunization[10], [11], and stochastic algorithms based on local network information, such as ring vaccination[12]–[14], acquaintance immunization[15] and community-bridge immunization[16]. Although studies have shown that deterministic immunization strategies regularly outperform stochastic immunization strategies in controlling the epidemics[16], the realistic global network information is usually or even constantly unavailable in the infectious diseases control practice. Therefore the stochastic immunization strategies and their optimizations have long been the research hotspots. However, the effectiveness assessment of the stochastic immunization strategies mentioned above is almost entirely made based on classical network models, such as scale-free networks[17] and small-world networks[18]. Although these network models have partly described the characteristics of realistic networks, there are still many differences among them, thus the real effectiveness of stochastic immunization strategies mentioned above has not been performed clearly.

In this article, the optimal immunization ratio 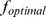, for acquaintance immunization and community-bridge immunization was determined and the influences of several primary factors on the effectiveness of ring vaccination, i.e., the case detection rate, the contact trace escape rate and the contact trace lag were analyzed based on the realistic urban social network of Portland. Then the effectiveness of these three different stochastic immunization strategies in controlling the spreading of infectious diseases were compared.

## Models and Methods

### Social contact networks

As the carrier of the diseases, daily activities of individuals underlie the spread of the epidemics in the population and people's choices about when and where to perform their activities are constrained by the transportation infrastructure[10]. Based on these assumptions and the sociodemographic data, researchers from Los Alamos National Laboratory (LANL) generate a synthetic population of Portland along with the detailed activity arrangement for each individual during one day. The obtained synthetic population data were applied into the TRANSIMS and EpiSims systems[10], [19]. The activities of the population during 24 hours and exacted the contact network of Portland were obtained from these data[20].

The final social contact network is an undirected weighted network with the individual attributes of the synthetic population recorded by each node and the contact duration of any two individuals in 24 hours recorded by the weight of the link between them. Moreover, the link between each pair of nodes has also recorded the contact type of these two nodes, involved nine different contact types. The nodes and edges attributes of the contact network were listed in Table 1.

**Table 1:** The nodes and edge attributes of the social contact network.

Node Attributes	Description	Edge Attributes	Description
Person Id	Id of the person	Duration	Duration of the contacts
Household Id	Household containing the person	Contact type:	
Age	Age of the person	0	Home
Gender	1 for Male, 2 for Female	1	Work
Household Size	Total number of people in the household	2	Shop
		3	Visit
		4	Social/Recreation
		5	Other
		6	Pick up or drop off a passenger
		7	School
		8	College

### SVIDR dynamics

**Figure 1:**
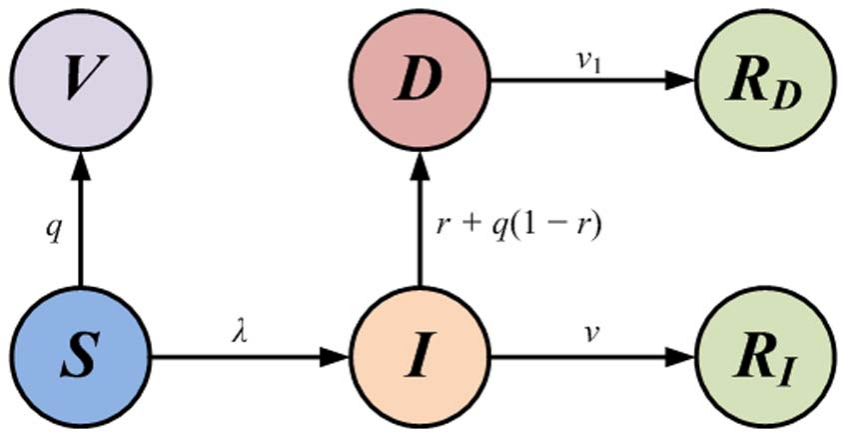
SIR state transition diagram with vaccination and isolation. The self-loop transition have been omitted. Class *V* and class *R* are absorbing states.

A simple extension of the SIR model[11], [21]–[23], will lead to the SVIDR model by adding three compartments, detected (*D*), vaccinated (*V*) and recovered detected (*R_D_*), see Figure 1. At any time, individual will be in and only in one specific state. A susceptible individual (*S*) suffers from an instantaneous infection rate 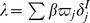 at time *t*, where 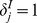 if *j* is in state *I*, otherwise 0. Therefore after a short time 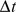, the probability of the individual being infected is 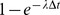. Susceptible individuals who are infected proceed to class *I*, and then will recover to *R_I_* at a rate 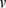. Alternatively, infectious individuals could be directly detected at probability *r*. Following direct detection, infectious individuals are moved into class *D* and recover to class *R_D_* at the a 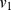, and meanwhile trigger the interventions. The interventions will dominate the probability of susceptible individuals proceeding to class *V* and this probability varies with interventions. Moreover, the probability of 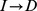 is also influenced by the interventions in addition to the direct detection probability *r*, which is called second-order detection (infectious individuals who escape the direct detection but captured by the interventions triggered by other individuals), see section 2.3. Individuals in class *V* and *R* (includes both *R_D_* and *R_I_*) will acquire fully immunity to the diseases and lose the susceptibility. Individuals in class *D* will be isolated and lose the capability to infect others.

### Immunization strategies

In practice of controlling infectious diseases, the global information of the contact network is almost constantly unavailable, thus highlights the importance of the stochastic immunization strategies based on local network structure. Three different strategies, i.e., acquaintance immunization (*AI*), community-bridge immunization (*CBI*) and ring vaccination (*RV*) were investigated. For *AI* and *CBI*, after the detection of the epidemics, the interventions were carried out across the whole social network. The only difference between the two strategies is that the nodes targeted to be immunized are different — *AI* targets high degree nodes while *CBI* targets community-bridge nodes. For *RV*, the interventions are implemented by tracing the contacts to detected nodes (*D*) and immunizing the traced nodes. From the perspective of the trigger mechanism and the intervention extent, *AI* and *CBI* are epidemic-triggered and global controlled strategies, while *RV* is newly confirmed case-triggered and local controlled strategy.

Denote the set of influenced nodes after the interventions were triggered by 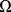, named controlled set. The controlled set consists of three different types of nodes, i.e., all the class *V*, *D* and partial class *R* nodes.

#### Acquaintance Immunization

**Figure 2:**
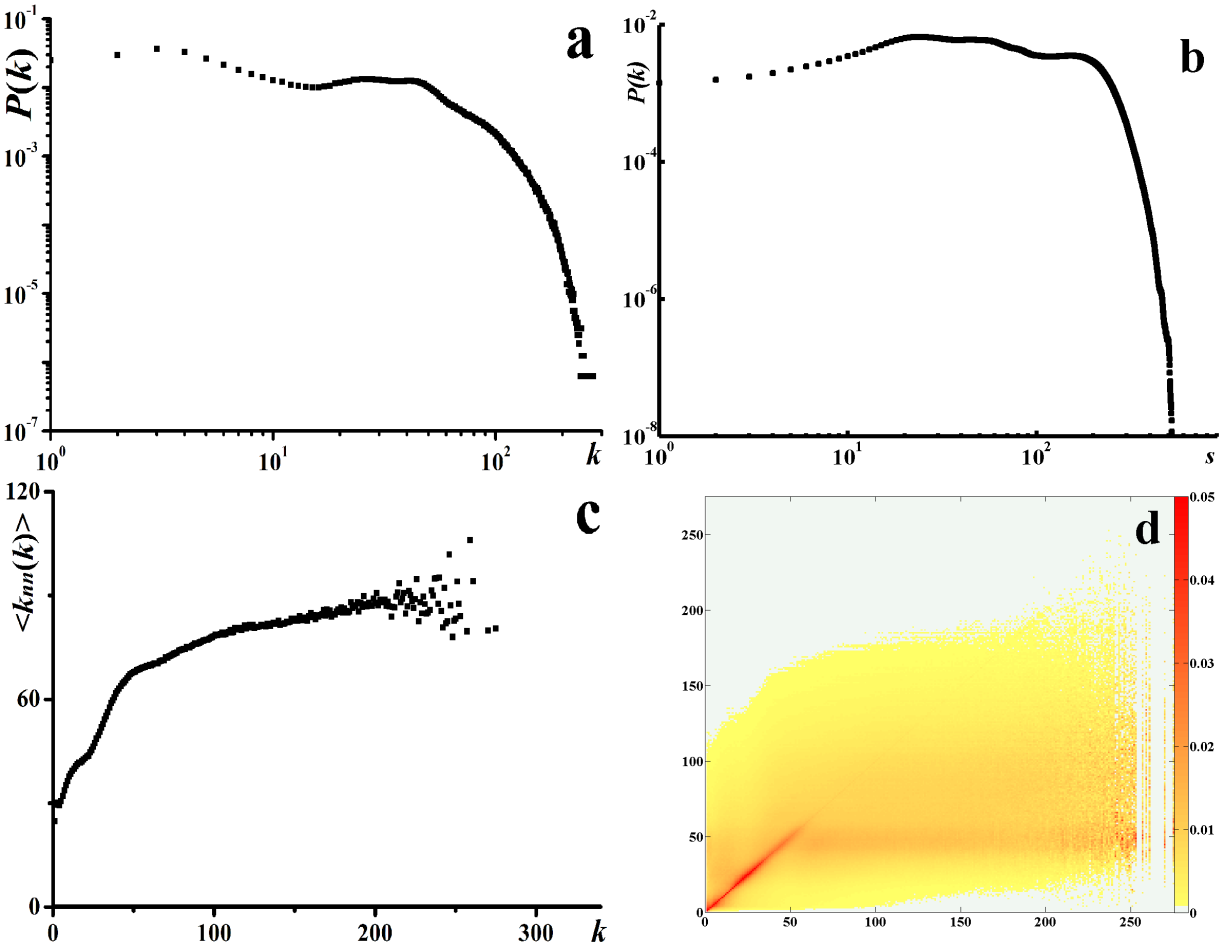
The topological characteristics of the social contact network. (a) log-log plot of the degree distribution. (b) log-log plot of the vertex strength distribution. (c) 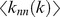 distribution. (d) heat map of 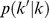.

*AI* applies to the scenario where large heterogeneity in contact structure is observed. The analysis of Portland contact network shows both the degree distribution and the vertex strength[24] distribution are highly heterogeneous (see Figure 2), so *AI* could be used to control the epidemics in this network. *AI* strategy is defined as follows: first, selecting *n* random nodes from the network; second, for each chosen node, immunizing a random neighbor. A node with *k* connections will be targeted as the immunization node with the probability 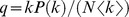 [15], where 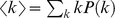 is the average degree. Therefore, for any susceptible individual, it will enter into the controlled set and proceed to class *V* with the probability *q*. For each infectious individual, it will enter into the controlled set and proceed to class *D* with the probability 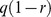 (second-order detection). Notice that the probability of direct detection is *r*, so class *I* individual will proceed to class *D* with the total probability 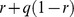. For individuals in other states (*V*, *R* and *D*), no influences were demonstrated on the spreading of the epidemics, so no processing was made.

#### Community-Bridge Immunization

Assume that a node will be targeted as the community-bridge node under the community-bridge find algorithm[16] with the probability *q*. The process of the targeted nodes is the same as it in *AI*, so it will not be repeated.

#### Ring vaccination

Suppose individual *j* has triggered an intervention, then the neighbors of node *j* in the contact network will enter into the controlled set with probability 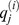, which varies with the contact type (*i*). The value of 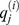 indicates the availability of the contact trace relate to type (*i*). For example, 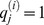
indicates that all the neighbors of *j* will enter into the controlled set, i.e., the contact trace escape rate related to type (*i*) is 0. Therefore, for every susceptible node, it will enter into the controlled set with the probability 
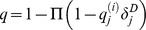
 and proceed to class *V*, where 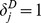 iff *j* is in class *D*, otherwise 0. For any infectious node, it will enter into the controlled set with probability 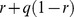 and proceed to class *D*. For individuals in other states, no other processing is required.

Let 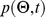 be the probability that individual is in class 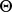 at time *t*. Assume that the above mentioned process is Markovian on the relevant time scales, the dynamics of this probability is governed by the following master equations:



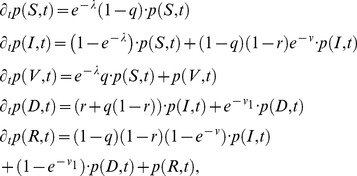
(1) where the computation of *q* varies with interventions.

## Results and Discussion

### Scenarios and parameterizations

At the beginning, 50 infectious individuals are seeded in the fully susceptible population. The fraction of class *I* individuals in the population is about 3.12 per 100000. In epidemiology, the basic reproductive number, 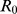 is defined as the number of cases one case generates on average over the course of its infectious period in an otherwise totally susceptible population[25]. 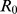 is one of the most important contributions of mathematics to epidemiology and it has provided a metric to evaluate the risk of the outbreaks of the epidemics in the population. Only infectious diseases with 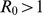
could possibly lead to a potential outbreak. The infectivity levels of infectious diseases may differ considerably. For example, for the pandemic influenza in 1918, 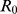 is between 2 and 3[26]; for SARS in 2003, 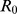 is between 1.8 and 4.2[27]; for H1N1 in 2009, 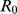 is between 1.8 and 3.2[28]; for smallpox, 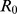 could reach 6.0[29]. Based on the analysis on the pandemic data from the 2009 H1N1 outbreak, 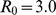, 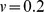 were chosen to parameterize our model for the baseline scenario in this research. To make the investigation more generally applicable and reliable, the scenarios when 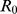 varies from 2.0 to 6.0 were analyzed in the sensitivity analysis. The relevant parameters are given in Table 2.

**Table 2:** The value of the parameters in the model.

Parameter	Baseline	Sensitivity analysis	Notes
*R*_0_	3.0	2.0–6.0	
*v*	0.2	N/A	
*v*_1_	0.25	N/A	The value of *v*_1_ doesn't affect the epidemic dynamic
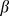	N/A	N/A	Parameterized from *R*_0_ and other relevant parameters: 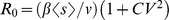
*f*	N/A	N/A	Parameterized by optimizing controlling the epidemics
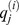 for *i* = 0∼1, 7∼8	1.0	N/A	For contact type 0, 1, 7 and 8, no contact trace escape
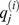 for *i* = 2∼6	0.7	0.3–1.0	For contact type 2–6, contact trace escape rate ranges from 0.3 to 1.0
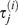 for *i* = 0∼1, 7∼8	0.0	N/A	For contact type 0, 1, 7 and 8, no contact trace lag
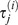 for *i* = 2∼6	1.0	1.0–3.0	For contact type 2–6, contact trace lag ranges from 1.0 to 3.0
*r*	0.6	0.3–0.9	In each day the probability of an infectious individual is detected (confirmed)

### Contact network structures

Portland social contact network consists of more than 31 million contacts of 1.6 million individuals during 24 hours. Each contact is represented by an undirected weighted link, where the weight denotes the contact duration in hours. The average degree is 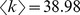, which is more than the precedent empirical results from questionnaire survey[30]. This difference is understandable because of the report of the respondents might miss some regular contacts and most random contacts. The comparison to the social networks from Facebook[16] shows that their average degree is close. The average clustering coefficient[18] is 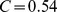 and the modularity[31] is 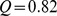. High heterogeneity was observed when taking the individual contact frequency (degree) into consideration, shown in Figure 2a. For weighted networks, the vertex strength[24] could be defined in addition to the degree: 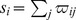. Figure 2b shows that the vertex strength distribution is also highly heterogeneous. The degree correlation could be measured based on the quantity 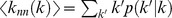, i.e., the average degree of the nearest neighbors of nodes with degree *k* [32]. Figure 2c shows that the slope of 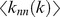 is positive, so the contact network is assortative mixing. The distribution of 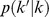 is given in Figure 2d.

### Optimizing immunization ratios

In this work, the optimization of *AI* and *CBI* was studied firstly. For *AI* and *CBI*, the immunization ratio *f* is defined as the fraction of the immunized susceptible individuals after the interventions are triggered, i.e., 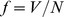. We then defined an objective function to investigate the optimal strategy. An intuitive and straightforward form of the objective function is given by number of individuals in class *R* and class *V* after the extinction of the epidemics, 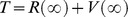. This definition follows the minimum affected principle and assigns equal weights to class *R* individuals and class *V* individuals[11]. Other more complex definitions could be obtained by weighting the two quantities.

**Figure 3:**
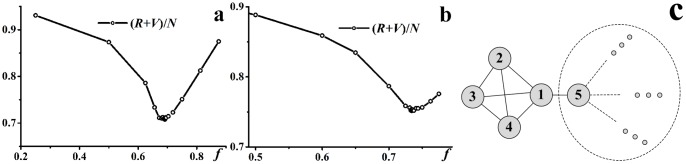
Optimal immunization ratio. 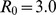
, 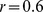. (a) 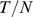 varies with *f* for *AI*, the optimal immunization ratio 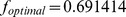. (b) 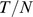 varies with *f* for *CBI*, the optimal immunization ratio 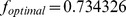. (c) A possible local network structure of contact network.

The binary search method was adopted to explore the optimal immunization ratio in interval [0, 1]. The search process ends when search step 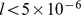. One notable thing is that the immunization ratio *f* for *AI* could be any value between 0 and 1: *f* = 0 indicates no interventions and *f* = 1 implies that all the individuals are immunized after the detection of the epidemics. However, for *CBI*, the immunized individuals are those targeted as community-bridge nodes by community-bridge find algorithm, which might be finite in the network. Take the sub network in Figure 3c as an example, according to the community-bridge find algorithm, node 1 and 5 are community-bridge nodes, i.e., potential immunization targets, while node 2, 3 and 4 are impossible to be targeted as immunization nodes. Therefore for any specific social contact network, there will be a ceiling immunization ratio 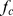 for *CBI*. 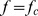 implies all the community-bridge nodes will be immunized. The realistic immunization ratio will always be less than 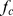.

Figure 3a and Figure 3b show the convergent trajectories of the optimal immunization ratio obtained by binary search method. For *AI*, a small *f* will lead to the unsuccessful control of the epidemics, while a large *f* will lead to a large amount of susceptible individuals immunized. 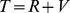 will display a “valley” shape and the optimal immunization 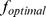 exists. For *CBI*, there is two possible different shapes of the convergent trajectory: one is the same as that in *AI*, where 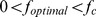; the other is the “slope” shape, which arises when 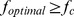, however, due to the finiteness of the community-bridge nodes in the network, the immunization ratio will be 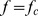.

### Analysis of key parameters of *RV*

*RV* is targeted locally in a ring around the identified sources of infection and is extensively investigated in theoretical studies on immunization strategies in responding to the outbreak of the smallpox and foot-and-mouth disease[12]–[14] as well as the disease control practice[33]. The success of *RV* depends on several crucial factors, such as the rapid identification of cases and the efficient contact trace of the identified cases, which could be captured by the following parameters: (i) the probability of diagnosis per day of infectious individuals, denoted by *r*; (ii) the probability of successful trace for the contacts of type *i*, denoted by 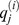; (iii) the time lag in tracing for the contacts of type *i*, denoted by 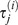. For close contacts, i.e., contacts in household, school and workplace, assume that the contact escape rate and the time lag are 0, that is, all close contacts could be traced and the immunization could be carried out immediately. The values of the parameters and related statements are listed in Table 2. For simplicity, the *q* was utilized instead of 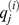 and 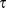 instead of 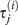 for casual contacts, where *i* = 2∼6.

**Figure 4:**
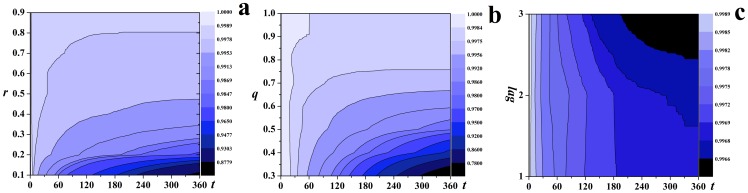
The effectiveness of *RV*. 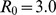
. The contour lines of the fraction of susceptible individuals with respect to time steps with (a) *r* ranging from 0.1 to 0.9; (b) *q* ranging from 0.3 to 1.0; (c) 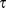 ranging from 1.0 to 3.0.

Figure 4 shows the dynamics of the epidemics varies with the parameters. With the increase of *r* and *q*, the contour lines of the fraction of susceptible individuals slope upwards, indicating that the effectiveness of *RV* are strengthened with the increase of the probability of diagnosis of class *I* individuals and the probability of successful traces for casual contacts, see Figure 4a and Figure 4b. Figure 4c shows the contour lines slope downwards, indicating that the increase of the time lag in tracing for casual contacts will lead to more affected individuals (individuals with states *R* or *V*).

### Effectiveness comparison and sensitivity analysis

**Figure 5:**
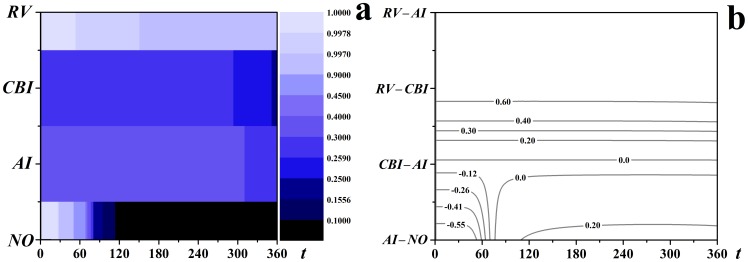
The comparison of the effectiveness of *AI*, *CBI* and *RV*. 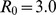
, 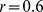, 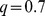, 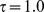. (a) the fraction of susceptible individuals varies with time steps with each strategy; (b) the differences of the fraction of susceptible individuals between each pair strategies.

The effectiveness of *AI*, *CBI* and *RV* in controlling the spread of infectious diseases has been compared. All these three immunization strategies were demonstrated to have successfully decreased the coverage of the epidemics compared to the scenario with no control measures (no control measures) in Figure 5a. A further comparative analysis of the difference in the fraction of susceptible individuals with respect to time steps between each pair of strategies were made in Figure 5b. Take the comparison between *AI* and NO as an example, the intersections between the horizontal line related to (*AI – NO*) and the contour lines show that (*AI – NO*) is negative in the early stage of the epidemics because of the dramatic decrease of the susceptible individuals resulting from the immunization after the outbreak of the diseases for *AI*. This phenomenon could also be observed in the scenario for *CBI*. With the evolution of the epidemics, the susceptible individuals will decrease more rapidly for *NO* than for *AI*, so (*AI – NO*) will become positive. The difference peaked around 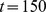 and then decreased, and was eventually positive, indicating that *AI* had decreased the coverage of the epidemics compared to *NO*. Similar analytics could be applied to other pair strategies (*CBI* – *AI*), (*RV* – *CBI*) and (*RV* – *AI*). Figure 5b shows *AI* outperforms *CBI* and *RV* significantly outperforms *CBI* and *AI* under the definition of the objective function 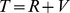.

**Figure 6:**
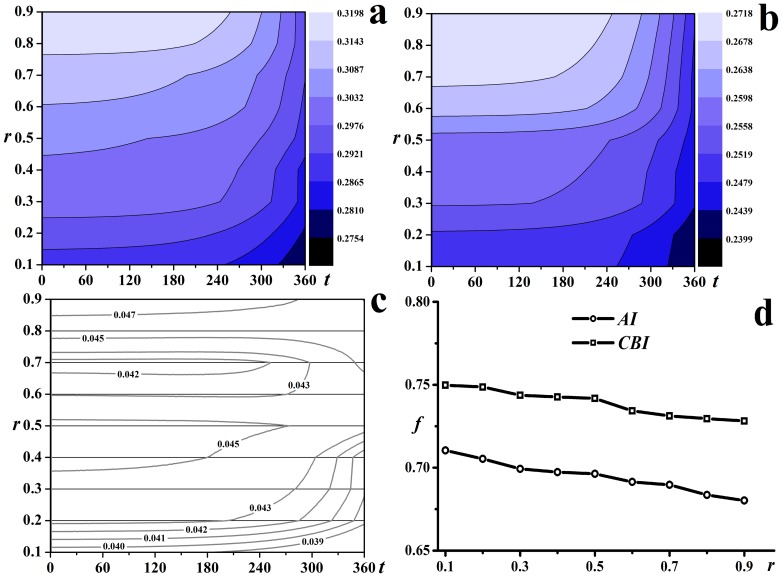
The effectiveness of *AI* and *CBI* with different *r*. The contour lines of the fraction of susceptible individuals with respect to time steps with *r* ranging from 0.1 to 0.9 for (a) *AI*, (b) *CBI* and (c) *CBI – AI*; (d) the optimal immunization ratios of *AI* and *CBI* with respect to different *r*.

The optimal immunization ratios for *AI* and *CBI* decrease with the increase of *r*, see Figure 6d. Figure 6a and Figure 6b give the dynamics of the epidemics with different *r*. Both of the contour lines of the fraction of susceptible individuals for *AI* and *CBI* slope upwards, indicating that the coverage of the epidemics decrease with the increase of *r*. The effectiveness of *AI* and *CBI* was further compared in Figure 6c, which shows *AI* always outperforms *CBI* for *r* ranging from 0.1 to 0.9 and the predominance will strengthen with the increase of *r*.

**Figure 7:**
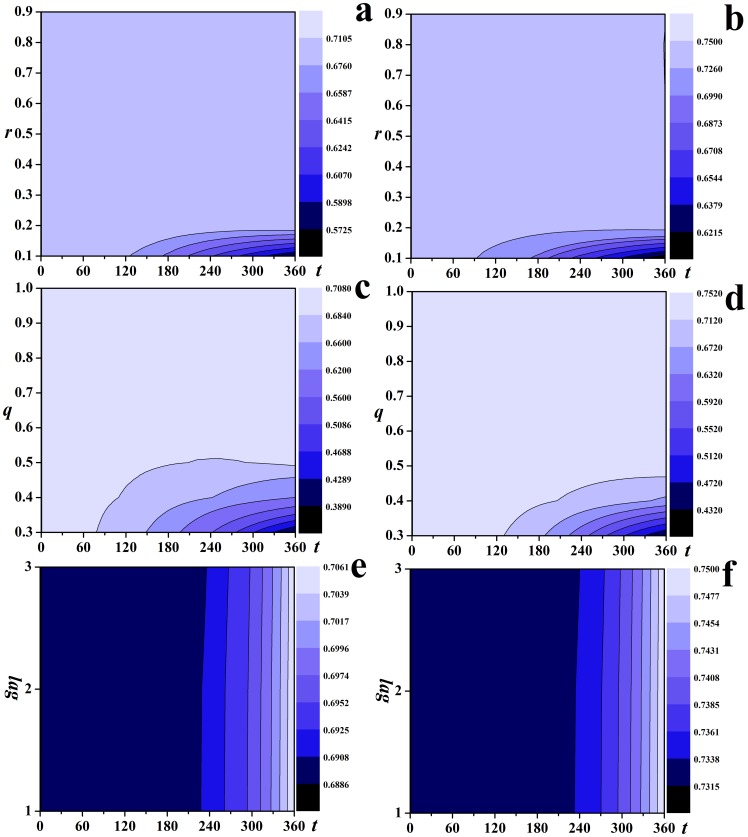
The effectiveness of *RV* compared to *AI* and *CBI* with different *r*, *q* and 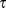. The contour lines of the fraction of susceptible individuals with respect to time steps for (a)(c)(e) *RV – AI* and (b)(d)(f) *RV – CBI*.

The effectiveness of *RV* is greatly denominated by *r*, *q* and 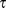. The effectiveness of *RV* compared to *AI* and *CBI* with different *r*, *q* and 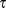 was analyzed in Figure 7. The contour lines in Figure 7a to Figure 7d slope upwards, indicating that the effectiveness of *RV* improves significantly with the increase of *r* and *q* compared to *AI* and *CBI*. It is notable that the dark colors are related to lower fractions of susceptible individuals, so the contour lines in Figure 7e and Figure 7f slope upwards slightly implies that the effectiveness of *RV* decreases with the increase of 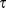, yet still significantly outperforms *AI* and *CBI*.

**Figure 8:**
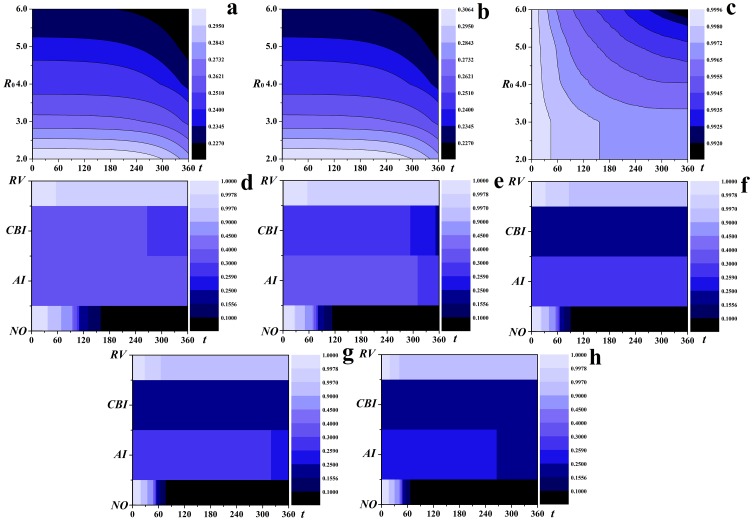
Effectiveness analysis of 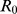 ranging from 2.0 to 6.0. The contour lines of the fraction of susceptible individuals with respect to time steps for (a) *AI*, (b) *CBI* and (c) *RV*; the fraction of susceptible individuals with respect to time steps with each strategy for (d)–(h).

Although recent large-scale outbreaks of the epidemics suggest 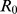 is usually between 2.0 and 4.0, studies on smallpox shows that its 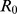 could reach 6.0[29]. Here the effectiveness of *AI*, *CBI* and *RV* for 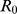 ranging from 2.0 to 6.0 was analyzed. Figure 8a to Figure 8c show the epidemic dynamics with different 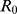. All the contour lines slope downwards, indicating the coverage of the epidemics increases with the increase of 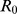 for all the three strategies. For *RV*, the declining rate of the contour lines decreases, indicating the number of susceptible individuals decreases more slowly. Therefore, *RV* is more effective compared to *AI* and *CBI*. More straightforward comparisons of the effectiveness of these three strategies with different 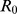 were given in Figure 8d to Figure 8h. With the increase of 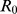, the affected individuals will increase for all the strategies, however, the decrease of susceptible individuals for *RV* is significantly slower than for *AI* and *CBI*, i.e., *RV* notably outperforms *AI* and *CBI*.

## Conclusions

In this paper the effectiveness of three stochastic immunization strategies in controlling the spreading of the epidemics based on realistic social contact networks was analyzed. We found that there exists an optimal immunization ratio for *AI* and *CBI* for a specific *r* which leads to a minimum number of individuals infected and immunized. This optimal immunization ratio decreases with the increase of *r* and could be determined by the binary search method. For *RV*, the case detection rate, the contact trace escape rate and the contact trace lag are three most important factors. The effectiveness of *RV* improves with the increase of the case detection rate, while decreases with the increase of the contact escape rate and the contact trace lag. The comparison of the effectiveness of these three strategies shows *AI*, *CBI* and *RV* have decreased the coverage of the epidemics compared to a baseline scenario (no control measures, *NO*), however the effectiveness of *AI* and *CBI* are very limited, between which *AI* outperforms *CBI*. *RV* is very effective in controlling the epidemics and its effectiveness significantly outperforms *AI* and *CBI*.

The sensitivity analysis shows the effectiveness of *RV* decrease with the decrease of case detection rate and the increase of contact trace escape rate and the contact trace time lag, yet still remarkably outperforms *AI* and *CBI* on equal terms. With the increase of the basic reproductive number 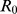, the coverage of the epidemics will increase for all these three strategies, however the increase of the number of class *R* and class *V* individuals for *RV* is much less than for *AI* and *CBI*, indicating that *RV* notably outperforms *AI* and *CBI*. Even when 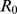 reaches 6.0, the effectiveness of *RV* is prominent, implying that *RV* could be applied to controlling the epidemics with a wide infectivity spectrum. We found that *RV* is a newly confirmed case-triggered and locally controlled strategy, so *RV* is more acceptable in infectious disease control practice, which will improve the effectiveness of *RV* in return. However, *AI* and *CBI* are epidemic-triggered and global controlled strategies. These control strategies call for large control resources due to a relatively high immunization ratio as analyzed in the main text, also the allocation and the logistics of the resources might be a problem. Moreover, the global control strategies might encounter the resistance of the public, so the effectiveness could be worse. Although the social contact network used in this research is just one instance for Portland urban population, it is the first time the effectiveness of several classical immunization stochastic immunization strategies are evaluated based on realistic contact networks. These results could have significant importance for epidemic control research and practice.
